# Book Review: Student Engagement in the Language Classroom

**DOI:** 10.3389/fpsyg.2021.713998

**Published:** 2021-06-18

**Authors:** Lingang Gu, Pingping Sun

**Affiliations:** ^1^Special Equipment Institute, Hangzhou Vocational and Technical College, Hangzhou, China; ^2^Business and Tourism Institute, Hangzhou Vocational and Technical College, Hangzhou, China

**Keywords:** student engagement, book review, student engagement in the language classroom, language classroom, learning environment

Although learner engagement has gained tremendous momentum in education (Reschly et al., 2020), it has received scant attention in language learning education. Therefore, *Student Engagement in the Language Classroom*, edited by Phil Hiver, Ali H. Al-Hoorie, and Sarah Mercer, aims to theoretically and empirically fill some gaps in L2 learning and opens up some innovative directions for future research, highlighting that L2 engagement is a pivotal condition for optimal learning.

The book is organized into two parts, preceded by an introductory chapter. The first part, encompassing four chapters, pertains to the definitional, conceptual, and measurement concepts about engagement. In the introductory chapter, the editors define engagement, relate it to the L2 classroom, and set the scene by outlining the following chapters. The authors in Chapter 2 aptly look at engagement from different dimensions: academic (task-based), affective, behavioral, cognitive, and social. They juxtapose engagement with investment, interest, and motivation, putting forward a continuous agenda for L2 engagement studies, aiming to reconcile theory and practice. Chapter 3 highlights a special type of engagement: *engagement with language* (EWL), focuses on how focus-on-form(s) can make learners meaningfully involved, and recapitulates some germane theories. Focusing on learner engagement with the written corrective feedback (WCF) at the micro and macro level of interactions is the objective of Chapter 4, proposing a critical standpoint at present research objectives. The last chapter in Part I highlights the role of the measurement of engagement. Synthetizing the literature on student engagement, not only do the authors argue that there is still some uncertainty on how engagement is operationalized and evaluated, but also they provide some avenues for further research about how learner engagement can be assessed in the L2 classroom.

Part II encompasses 10 chapters that empirically scrutinize L2 learner engagement from different vantage points. For instance, Chapter 6, set in the French context, concludes that the learning environment has a decisive role in L2 classroom engagement since the interconnectedness of peers, teachers, and learning tasks can interactively impact learners' involvement. Set in the Spanish context, Chapter 7 explores the role of face-to-face and synchronous computer-mediated communication in the affective, behavioral, and cognitive engagement of L2 students, concluding that mode of task-based negotiations can differentially engage learners.

Chapter 8 seems genuinely appealing to me because of its exploratory nature of engagement from the learners' viewpoints. This chapter reports that sometimes learners deliberately manipulate their conduct to pretend involvement to mask disengagement and fulfill social expectations in the L2 classroom. The authors judiciously remark that we need to be cognizant of overreliance on observable classroom behaviors, and we should not consider them as an unproblematic enterprise for considerable engagement in learning. Focusing on the emotional engagement and embarking on the mixed-methods data, Chapter 9 unravels how the impact of action choice and options choice through both negative and positive emotions can contribute to varying manifestations of emotional engagement, performance, and task focus.

Important is the exploration of *prosocial engagement*, defined as “as a type of engagement in which students' perceptions of their mutually constructive participation matter to them in their academic achievement” (p. 182), as an innovative approach to learner engagement. The authors, in Chapter 10, persuasively endorse that prosocial engagement is influential to fight learners' passivity, disengagement, and reluctance to involve students based on their mind–time frames that can be instantiated in prosocial engagement. Chapter 11 is an outstanding contribution in this volume that follows a theory meets-practice approach and scrutinizes deeply how immersion in virtual contexts can focus on all dimensions of student' involvement. The chapter specifically highlights the advancement of a virtual reality project whose focus is to reconstruct an authentic, experiential, and game-like milieu, which can foster social negotiation.

Set in the Japanese context, Chapter 12 draws on the longitudinal study to elucidate how young learners made substantial improvements in cognitive engagement in the language classroom. Rarely explored in the Iranian context is the role of positive and negative L2 classroom emotions and grits in the reading comprehension classroom. In Chapter 13, the author examines how classroom emotions and grits are predictive of L2 engagement, and how L2 engagement relates to learners' L2 reading comprehension. In Chapter 14, set in the German context, the authors embark on the longitudinal case study to showcase that learner's willingness to engage (WTE) is an indispensable antecedent situation that materializes before the students” actual involvement. The authors argue that success in L2 learning is contingent upon the proactivity and involvement of learners. Finally, Chapter 15 closes this volume by recapitulating the results of the preceding chapters and outlining some avenues for future research.

This thought-provoking compendium enjoys many merits. First, the contributions in this volume are from diverse geographical locations, indicating the sagacious selection of the editors who are cognizant of the context-specific, culture-bound nature of L2 engagement. Secondly, this volume has done its best to reconcile theory and practice by foregrounding the theoretical postulations and pedagogical implications of these robust, innovative, and diverse data-driven studies in language learning. Thirdly, the empirical studies have utilized diverse methodological designs, rigorous data collection instruments, innovative concepts, making this volume a substantive resource for the readers. Finally, the volume has focused on L2 engagement not only at the school level but also at the tertiary and higher education level. However, had the editors included studies about the impact of administrative staff on learner engagement, it would have been insightful.

In sum, we feel confident to recommend this theoretically robust and empirically insightful compendium as a treasure chest of resources to language students, language learning theoreticians, researchers, practitioners, teachers, syllabus designers, and policymakers who will find this collection a rich and contemporary addition to the literature on L2 learner engagement.

## Author Contributions

All authors listed have made a substantial, direct and intellectual contribution to the work, and approved it for publication.

## Conflict of Interest

The authors declare that the research was conducted in the absence of any commercial or financial relationships that could be construed as a potential conflict of interest.
